
JOURNAL INFORMATION
==============================
Journal ID: ijcoms

IJQHC Communications

EISSN: 2634-5293

ARTICLE INFORMATION
==============================
PMCID: PMC8574486
DOI: 10.1093/ijcoms/lyab017
Article ID: lyab017
Article version: 1
Subjects: Perspective, AcademicSubjects/MED00862

Covid-19 – A Game Changer for Medical Education? Reflections from England

Vaux Emma Royal Berkshire NHS Foundation Trust - Berkshire Kidney Unit, Royal Berkshire Hospital RG1 5AN, Reading, United Kingdom
Royal College of Physicians - Assessment Unit, London, United Kingdom

Moss Fiona London, United Kingdom

Corresponding author: Emma Vaux, Royal Berkshire NHS Foundation Trust - Berkshire Kidney Unit, Royal Berkshire Hospital RG1 5AN, Reading, United Kingdom, tel: +447788780214; email: emma.vaux@royalberkshire.nhs.uk
Electronic publication date: 2021 Oct 27
Electronic Location ID: lyab017
Received 2021 May 20; Revised 2021 Aug 24; Accepted 2021 Oct 25; 2021 Oct 14
Copyright: © The Author(s) 2021. Published by Oxford University Press on behalf of International Society for Quality in Health Care.
Copyright year: 2021
License: This is an Open Access article distributed under the terms of the Creative Commons Attribution License (https://creativecommons.org/licenses/by/4.0/), which permits unrestricted reuse, distribution, and reproduction in any medium, provided the original work is properly cited.
License URL: https://creativecommons.org/licenses/by/4.0/

edited-state: accepted-manuscript
article-lifecycle: PAP

==============================

TITLE PAGE

**Covid-19 – a game changer for Medical Education**

An invited commentary

Emma Vaux[1,2] and Fiona Moss[3]

1 Royal Berkshire NHS Foundation Trust - Berkshire Kidney Unit, Royal Berkshire Hospital Reading RG1 5AN, Reading, United Kingdom

2 Royal College of Physicians - Assessment Unit, London, United Kingdom

3 London, United Kingdom

Corresponding author:

Emma Vaux

Royal Berkshire NHS Foundation Trust - Berkshire Kidney Unit, Royal Berkshire Hospital Reading RG1 5AN, Reading, United Kingdom tel: +447788780214

email: emma.vaux@royalberkshire.nhs.uk

ACCEPTED MANUSCRIPT

**Covid-19 – A Game Changer for Medical Education? Reflections from England**

Prior to Covid-19 many medical educators grappled with a host of challenges such as changing expectations of patients and the public; new approaches to care delivery; and the rapid growth of medical technologies. The 4th industrial revolution ushered in the emergence of genomics, nanomaterials, and applications of artificial intelligence, robotics, and data science into the healthcare space. These innovations have enhanced both diagnostic and therapeutic capabilities and likewise changed the content of medical education.

Many institutions of higher learning had already embraced changes in training in response to these new technological advances, such as access for the University of Warwick medical students to the Institute of Digital Healthcare (1). The focus for future medical practice was how to leverage technology to enhance the partnership between patient, physicians, and other healthcare providers

© The Author(s) 2021. Published by Oxford University Press on behalf of International Society for Quality in Health Care.

(HCPs), to maximise all contributors to care delivery. Innovations in early 2020 included: inter-professional board rounds, in-situ simulation, digital tools (2) and flexible portfolio training (3). But the changes to education have not been as rapid or as profound as the changes in technology.

The Covid-19 pandemic has acted as a massive disruptor to the delivery of health care and health care education prompting sweeping changes implemented at pace. Many HCPs, including doctors in training, have experienced fundamental changes in what they do, in how they work and in their education. Throughout, some trainees have had exceptional training and learning opportunities but for most education has been compromised: educational activities cancelled, regular changes in rotations affected and many missed core rotations. Teachers and trainers have had to support and improvise in ways that they would never have imagined eighteen months ago. The experience accrued is unique and perhaps gives an insight into how we should learn and train in the modern health care environment.

An area of crucial importance is essential clinical teaching. Developing good clinical skills is the basis of medical education and good medical practice. Restrictions and preventative measures have prompted the emergence of different types of virtual care. Of course, emails and video consultations are not the same as in-person experience, but for many patients virtual encounters may be better and more efficient than in-person ones. Deployment of virtual care with an appropriate balance with in-person care is likely to continue. For medical education this has three significant implications. First, it will be important to consider how best to teach about clinical signs, symptoms and presentations in the context of current modes of care delivery. Secondly, virtual consultations are easier for the experienced practitioner than the trainee and may require different approaches to supervision. Thirdly, undertaking a virtual consultation will be an important competency that will need to be taught and assessed. These are just some of the ways that the "how, what, where and why" we teach basic clinical skills will need to change quite significantly in the next few years in response to the changes to how clinical medicine is practiced. Meanwhile, the health care response to the pandemic has also highlighted the central importance of the organisation of care and the importance of the link between organisational and clinical practice for good care.

The experiences of the pandemic may help integration of the skills needed for quality improvement (QI) into mainstream education. QI has been part of the lexicon of care for about 30 years. Despite some beacons of practice, the implementation of QI has been somewhat incremental and genuine integration into education has been slow too. Organisational change is a key factor to QI: all improvement requires organisational change and by implication changes to the way people work. But through the pandemic, all HCPs, including current trainees, now have lived experience of radical organisational change. Now is the time to teach all HCPs - including both trainees and experienced practitioners - in the range of organisation skills [4,5] needed for QI whilst the experience of significant organisational changes required to manage the challenge of Covid-19 is fresh and so more likely to become embedded in routine practice.

Team work has been so palpably at the core of the response to the pandemic. We have always known that teams and team working are important: good care is the production of functional teams and not individuals (6). Team skills are included in curricula but recognised more in theory than in practice. The staging points of medical education are based almost exclusively on the assessment of individual acquisition competencies and not about team competencies: it is possible for a group of individuals with proven individual competencies who when they come together perform incompetently as a team (7). Health care teams are by nature multi-professional. A genuine concern about development of team competences and skills will require more collaboration between the

ACCEPTED MANUSCRIPT

educators of different health care professions. In addition, learning about team skills should be a matter for career long continuing professional development as about speciality training.

None of this is new – the importance of organisational skills and team working have been acknowledged for some time, but getting ideas into educational practice has been slow. Health Education England's "The Future Doctor Programme" published during the pandemic started consultation in 2019 in response to NHS England's "NHS Plan" and included a review of the themes relating to the future clinical team (8). It is a blue print that needs now to link with other professions' training programmes and to be integrated into primary care networks and into hospital trusts/health boards. Every specialty needs to have its curriculum; each profession its own approach to training but all the educational systems need to come together at the point of practice to ensure that the organisational skills that have been so essential for the NHS response to the pandemic are retained and developed and become part of the dailiness of care. Connectedness of teams and health systems should be at the core of care delivery in the NHS: bringing together education systems will be key.

Delivery of care during the pandemic has shown us that no one set of principles, training pathway, guidelines or algorithm stands alone. The experience of the pandemic may have demonstrated how the education and training of HCPs could be modernised and may have enhanced some aspects of training for some. Flexibility in how we train is high on the agenda and robust processes are now in place in the UK to allow transferability of professional capabilities between specialties (9). Another area of spotlight is the how we enhance generic learning between specialties and HCPs in areas such as social determinants of health and learning disability, crucial if the health inequity gap is to be narrowed. There is no doubt though that for others their expected progress has been markedly slowed. For example, for surgical and other craft specialities trainees, there has been a significant reduction in the opportunity for learning procedural skills. Current trainers will be working to support them and many may have to extend their training. The impact of infection control has slowed procedures and continues to be a further barrier to access to operative training for some. Finding ways to enhance operative training including increased use of simulation, haptics and other virtual approaches to training is an important and urgent issue.

The pandemic has prompted both the acceleration of many changes already intended to address the educational needs of 21st century practice and the creation of opportunities not previously imagined. Blended learning in hybrid environments, alternative assessments, and the enhanced use of technology as training tools are just some of the changes that will contribute to the education of all future HCPs (10). Continued resilience, flexibility and convergence of both our educational and health care delivery systems will be essential, using the experience of delivering care and teaching through the pandemic, to take forward the structural reforms so necessary if education is to keep pace with both advancing technologies, changes in the delivery of care, and changing patient expectations.

We have, through the insights provided by the "whole service" response to Covid-19, a singular opportunity to respond to the many learning gaps and the inequities and inequalities in health, by putting education and training at the heart of what we do. This is not a matter for the future: changes need to be put into practice now with current HCPs and for today's patients. Ensuring that HCPs not only learn about the scientific, technical and clinical aspects of care but are also equipped to collaborate, to work effectively within teams and implement changes for improvements in the quality and safety of care locally, would be a very fitting memorial to the many HCPs who have died from Covid-19. The response to the pandemic has shown just how much can be achieved: the work now is to make sure that that learning endures.

ACCEPTED MANUSCRIPT

**REFERENCES**

1. Institute of Digital Healthcare, University of Warwick https://warwick.ac.uk/fac/sci/wmg/idh/about/ [Accessed March 2021]
2. Royal College of Physicians. *Never too busy to learn: How the modern team can learn together in the busy workplace.* www.rcplondon.ac.uk/projects/outputs/never-too-busy-learn-how-modern-team-can-learn-together-busy-workplace [Accessed 30 March 2021]
3. Royal College of Physicians. *Flexible Portfolio Training.* https://www.rcplondon.ac.uk/projects/flexible-portfolio-training [Accessed 30 March 2021]
4. Swanwick T, Vaux E. *ABC of Quality Improvement in Healthcare (ABC Series)* London: Wiley-Blackwell Publishing; 2020
5. Training for better outcomes Developing quality improvement into practice. https://www.aomrc.org.uk/reports-guidance/developing-quality-improvement-into-practice/ Academy of Medical Royal Colleges [Accessed 30 March 2021]
6. Lyuobvnikova, J., West, M., Dawson, J. & Carter, M. 24-Karat or fool's gold?: Consequences of real team and co-acting group membership in healthcare organizations. *European Journal of Work and Organizational Psychology*. 2015; 24, 6, 929-950
7. Lindgard,L. *KeyNote Lecture:International Conference Residency Education* https://www.youtube.com/watch?v=X7PJqHfhkMQ [Accessed 30 March 2021]
8. Health Education England. *The Future Doctor Programme.* https://www.hee.nhs.uk/sites/default/files/documents/Future%20Doctor%20Co-Created%20Vision%20-%20FINAL.pdf
9. Academy of Medical Royal Colleges. *Guidance for flexibility in postgraduate training and changing specialties* https://www.aomrc.org.uk/wp-content/uploads/2020/06/Guidance_flexibility_postgraduate_training_0620.pdf [Accessed 30 March 2021]
10. Royal College of Physicians. *Never too busy to learn – a pandemic response 2020* https://www.rcplondon.ac.uk/projects/outputs/never-too-busy-learn-pandemic-response-0 [Accessed 30 March 2021]

ACCEPTED MANUSCRIPT
